# Effect of Moxibustion on Testosterone Secretion and Apoptosis of Spermatogenic Cells in Aging Rats

**DOI:** 10.1155/2019/5186408

**Published:** 2019-10-24

**Authors:** Chongjie Yao, Chen Zhao, Shuyan Zhang, Shimin Liu

**Affiliations:** ^1^Yueyang Hospital of Integrated Traditional Chinese and Western Medicine, Shanghai University of Traditional Chinese Medicine, No. 110 Ganhe Road, Hongkou District, Shanghai 200437, China; ^2^College of Acumox and Tuina, Shanghai University of Traditional Chinese Medicine, Room 9405, No. 1200 Cai Lun Road, Pudong New District, Shanghai 201203, China

## Abstract

Analysis of androgen secretion and sperm production was conducted in the testis to investigate the efficacy of moxibustion on testicular function in aging rats. Male Sprague–Dawley rats were randomly divided into the aging group (*N* = 8), the mild-warm moxibustion group (*N* = 8), and the youth control group (*N* = 8). Rats in the mild-warm moxibustion group (MWMG) were exposed to mild-warm moxibustion at the Zusanli (ST36) and Shenshu (BL23) acupuncture points daily, from the age of 12 months until the age of 24 months. After the intervention, testicular tissue was harvested from all rats across groups. Changes in testicular structure were examined by hematoxylin and eosin (H&E) stain. Detection of the apoptosis of spermatogenic cells was performed by the TUNEL assay. Testosterone level in the testis was analyzed by the ELISA assay, and the expression of Bax, Bcl-2, and androgen receptor (AR) in the testis was evaluated by immunohistochemistry. AR expression analysis was subsequently performed by the western blotting assay, and the detection of telomerase activity of the testis and the expression of Bax, Bcl-2, and AR mRNA were performed by real-time PCR. Compared with the youth controls, telomerase activity in the testis, testosterone levels, expression of AR, and expression of antiapoptosis factor Bcl-2 protein and mRNA were significantly decreased (*P* < 0.01) in the aging group. Spermatogenic cell apoptosis (*P* < 0.01) and proapoptotic factor Bax expression were significantly increased (*P* < 0.01) in the aging rats compared with the youth control group. The MWMG exhibited significant increases in testicular telomerase activity, testosterone level, AR expression, and antiapoptosis factor Bcl-2 expression (*P* < 0.05 or *P* < 0.01) compared with the aging group. In this experimental group, spermatogenic apoptosis was inhibited (*P* < 0.01) and proapoptotic factor Bax expression significantly reduced (*P* < 0.01). Mild-warm moxibustion can inhibit reproductive senescence by improving telomerase activity, improving AR expression, restoring testosterone, and inhibiting spermatogenic apoptosis via regulation of Bcl-2/Bax.

## 1. Introduction

Generally speaking, senescence refers to the gradual decline of physical fitness and is linked to the increased risk of illness and death. As a natural process, senescence is subject to the impacts of hereditary, epigenetic, and environmental factors and is reflected at cellular and molecular levels [1, 2]. As the aging population continues to grow, delaying senescence has become an increasingly interesting research topic and is considered of great importance to the general population [3]. The issue of senescence has been explored by traditional Chinese medicine since time immemorial. “The Yellow Emperor's Classic of Medicine,” an ancient treatise on health and disease believed to have been authored by the Chinese emperor Huangdi c. 2600 BC, describes that the senescence of a biological organism is based on reproductive senescence. This theory has been largely corroborated by modern research indicating that testicular degeneration is closely related to the senescence of biological organism and may be used as an indicator of overall senescence [4].

Confirmed by clinical studies, age-related testosterone (T) declines are associated with clinical signs and symptoms such as sexual dysfunction, emotional changes, loss of muscle, and evident increases in the likelihood of cardiovascular and all-cause mortality. Thus, optimizing testicular function in elderly males may be vital for maintaining their health [5, 6]. Currently, hormone therapy is accepted as the mainstream treatment; however, the clinical efficacy and side effects of testosterone supplementation therapy remain controversial, and the importance of alternative treatment is self-evident [7].

As a traditional Chinese medicine technique, moxibustion has been widely applied across different types of diseases including gastrointestinal diseases, gynecological diseases, and various sorts of pain. Currently, the biological effects of moxibustion have been evidenced by genetic analysis by the researchers which have identified its role in key processes such as translation and transport of key mRNAs in aging rats [8]. Moxibustion has also exhibited antiaging effects, protection of neuronal structure, and reduction in the rate of neural degeneration [9]. Previously, this team has studied the effects of moxibustion on ovarian granulosa cell apoptosis in aging rats, elucidating a mechanism for improving perimenopausal syndrome in female rats by [10]. To date, few studies have addressed whether moxibustion can improve testicular function and its underlying mechanisms.

This study aimed to investigate the efficacy of moxibustion on testicular function in aging rats. This efficacy was analyzed from the perspectives of androgen secretion and sperm production in the testis, which may be correlated to the alteration of testicular structure, changes in telomerase activity and androgen receptor (AR) expression, as well as apoptosis. The results of this study indicate that moxibustion can increase T levels, improve testicular structural damage caused by aging, enhance telomerase activity and AR expression, and inhibit the apoptosis of spermatogenic cells, ultimately highlighting an alternative strategy for improving testicular function.

## 2. Materials and Methods

### 2.1. Animals

Eight immature male Sprague–Dawley rats (3-4 weeks) and sixteen adult males (12 months, weight 700 ± 50 g) were purchased from Shanghai BiKai Experimental Animal Co., Ltd. (certificate number: SCXK (Shanghai) 2013-0016) and housed in the Laboratory Animal Unit of the Shanghai University of Traditional Chinese Medicine. All the rats were housed in standard laboratory conditions (22∼27°C, 50–70% indoor humidity) under a 12 h light-dark cycle with rat chow and water *ad libitum* for one week before the experiment. To alleviate pain and avoid injury, animal feeding, care, and all experiments were carried out in accordance with procedures approved by the Experimental Animal Ethics Committee of the Shanghai University of Traditional Chinese Medicine. All protocols were strictly implemented in accordance with the “Guidelines for the Protection and Use of Experimental Animals” formulated by the Ministry of Science and Technology of the People's Republic of China. Every effort was taken to minimize animal suffering.

### 2.2. Grouping and Treatment

Sixteen adult male Sprague–Dawley rats were randomly divided into the aging group and the mild-warm moxibustion group (MWMG) (*N* = 8). In addition, 8 immature male rats comprised the youth control group. Rats in the MWMG began moxibustion at the Zusanli (ST36) and Shenshu (BL23) acupuncture points from the age of 12 months. Moxa sticks 5 mm in diameter were ignited 2 cm above the acupuncture points of interest for 20 minutes. The acupuncture points on either side were treated on alternate days, and the procedure was performed daily until the rats were 24 months of age. Rats in the aging group (*N* = 8) and the youth control group (*N* = 8) did not receive any treatment but received equivalent frequency of grasping and fixation. After treatment of the MWMG was completed, the rats in all three groups were euthanized and testicular tissues were harvested. The external fascia of testis was peeled back, and the testis was rinsed with normal saline. Tissues were then hemisectioned, and one half was dehydrated and paraffin embedded after 48-hour fixation in a 4% paraformaldehyde solution. The remaining section was cryopreserved at −80°C for molecular assays.

### 2.3. Observation of Testicular Structure

Paraffin-embedded sections were cut into thin slices (4 *μ*m) and deparaffinized. Changes in testicular structure were evaluated under the microscope at 100x magnification after hematoxylin and eosin (H&E) staining.

### 2.4. Apoptosis of Spermatogenic Cell Detection by TUNEL Assay

Paraffin-embedded sections were cut into thin slices (4 *μ*m) and deparaffinized. Nuclei were processed according to the manufacturer's instructions of a TUNEL assay kit (BOSTER Biological Technology Co., Ltd.). Nuclei were then counterstained with hematoxylin. The total number of TUNEL-positive nuclei and spermatogenic cells were counted under a microscope at 200x magnification with the assistance of ImageProPlus software. The ratio was recorded as the apoptotic index (AI) of the spermatogenic cells. Five fields were randomly selected from each slice to calculate AI, and at least 100 cells were counted in each field.

### 2.5. Testicular Testosterone ELISA Assay

Frozen testicular tissues were cut into thin sections and homogenized; the cells were then lysed thoroughly in RIPA Lysis Buffer (Beyotime) at a concentration of 10 *μ*l/mg of tissue, until the tissue pieces dissolved in the lysis buffer. The cell lysate was centrifuged at 12,000 ×g for 5 minutes, and the total protein of the supernatant was calculated by using a BCA protein concentration assay kit (BOSTER Biological Technology Co., Ltd.). The testosterone level in the testis was measured using a sandwich ELISA according to the manufacturer's protocol provided for the testosterone ELISA kit (BOSTER Biological Technology Co., Ltd.). Ninety-six well plates were used for each sample, which was tested in duplicate to improve accuracy. After 50 *μ*l tissue homogenate was added to each well, 50 *μ*l of an enzyme-labeled antigen and 50 *μ*l of antibodies were added, completely mixed, and incubated at 37°C. These were washed thrice after 1 hour. Fifty microliters of color-developing agent A and 50 *μ*l of color-developing agent B were mixed and added into each well; the color was developed at 37°C for 15 min, following which 50 *μ*l of stop solution was added. The absorption spectra for proteins were 450 nm. The ratio of testosterone to total protein content (ng/mg) of the tissue was calculated by the total protein concentration of the sample.

### 2.6. Immunohistochemical Detection of Bax, Bcl-2, and AR Expression

Paraffin-embedded sections were cut into thin slices (4 *μ*m) and deparaffinized. Antigen retrieval was achieved after the sections were boiled in citric acid buffer for 20 seconds and cooled to room temperature for 1 hour. Subsequently, the sections were incubated in 1% H_2_O_2_ solution for 25 min to block endogenous peroxidase activity, followed by rinse with distilled water. Sections were then incubated in 5% BSA at room temperature for 20 minutes after drying, followed by primary antibody incubation (Bcl-2, 1 : 200, Abcam; Bax, AR: 1 : 500, Abcam) overnight at 4°C. The following day, sections were rewarmed in an oven at 37°C for 45 minutes and incubated in goat antimouse/rabbit IgG for 20 minutes. Next, SABC solution was added for another 20 minutes. After rinsing, the sections were treated with diaminobenzaidine (DAB) and counterstained with hematoxylin. Finally, the sections were mounted and slides were soaked in 1% hydrochloric acid for 3 seconds, dehydrated step-wise with ethanol, stripped by xylol, and finally sealed with Neutral Balsam. Five fields of view were randomly selected from each specimen and evaluated under a light microscope at 100x magnification with the aid of ImageProPlus software. The values of integral optical density (IOD) were determined by the pale brown IHC presentation indicating immunopositive tissues in each field of view. The mean optical density (MOD) of each specimen was calculated and collected after total areas (Area) were measured.

### 2.7. Detecting AR Expression in Testis by Western Blotting Assay

The extraction protocol for total protein quantitation of testicular tissue was described above in Section 2.5. After adjusting the protein concentration, 20 *μ*g protein per sample was dissociated from a 12% SDS-PAGE gel and transferred to a PVDF membrane. The membrane was incubated with a primary antibody (AR, 1 : 1000, Abcam) overnight at 4°C after being blocked by 5% BSA (bovine serum albumin) blocking buffer at room temperature for 2 hours. After washing of the membrane, the sample was incubated with HRP-labeled secondary antibody (1 : 2000, Jackson) at room temperature for 2 hours and the membrane was washed. The membrane was put into the color box in a dark room, and mixed color-developing agent ECL was added (reagent A: *B* = 1 : 1, BOSTER Biological Technology Co., Ltd.). After reaction for 2 minutes, the sensitive film was placed on the membrane and the membrane was put into the X-ray radiographic cassette to be exposed for about 5 minutes; the time depends on the degree of the exposure. The film was then rinsed in a developer solution until the strip was clear and rinsed in the fixer solution for about 10 seconds until the film was transparent. Finally, the images were scanned; ImageJ software was used to calculate the gray value ratio of objective protein/internal reference protein *β*-actin.

### 2.8. Detection of Bax, Bcl-2, and AR mRNA

Testicular tissue (0.1 g) was used, and its total RNA was extracted using the Trizol method. Subsequently, 1 *μ*l of total RNA was added to a 0.2 ml PCR tube for RNA reverse transcription and then incubated at 42°C for 60 min and 72°C for 10 min. The cDNA template was obtained as the product, which was used after dilution of the cDNA. After adding 5 *μ*l of the SYBR Green mix (Beyotime), 0.4 *μ*l of upper primer and 0.4 *μ*l of lower primer to 2 *μ*l of cDNA and RNase-free H_2_O was added to constitute 10 *μ*l and then mixed thoroughly. The reaction conditions were set for PCR amplification. With *β*-actin as the internal reference, each sample was added into triplicate wells, and the quantitative fluorescence data collected were analyzed. The final results were analyzed using the relative quantitation method, i.e., the 2^−△△CT^ value. The primers used are shown in Table 1.

### 2.9. Detection of Testicular Telomerase Activity by PCR Telomerase Assay

cDNA obtained in Section 2.8 was processed according to the instructions of a PCR telomerase detection kit (Jiangsu KeyGen BioTECH corp., Ltd.). Telomerase activity was detected indirectly by the mRNA expression level of a catalytic subunit of  telomerase, termed telomerase reverse transcriptase (TERT).

### 2.10. Statistical Analysis

SPSS 21.0 software was used for the analysis. The normality and homogeneity of variance tests were performed on the data. The data were presented as the mean ± standard deviation (±s), if they were normally distributed. Data that did not conform to the normal distribution were presented as the median (*P*25 and *P*75). If the data conformed to the normal distribution, the comparison of differences among groups was performed using one-way ANOVA. If the variances were homogeneous, a pairwise comparison was performed using the least significant difference (LSD) test. If the variances of all the groups were not homogenous, a nonparametric Kruskal–Wallis *H* test for the analysis and pairwise comparison, using the Nemenyi method, were performed. If the data did not conform to the normal distribution, a rank sum test was performed. The significance level of the statistical examination was *α* = 0.05. Therefore, *P* < 0.05 indicated a significant difference.

## 3. Results

### 3.1. Moxibustion Improved Testicular Structure

**Figure 1:**
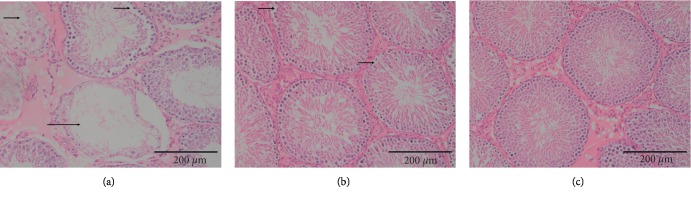
Rat testicular structure observation (bar = 200 *μ*m). (a) Aging group: the spermatogenic cells were distributed loosely and disorderly, part of the structure disappeared, spermatogenic cells and Sertoli cells were reduced apparently, part of the spermatogenic epithelium shed, and the seminiferous tubules were atrophied and necrotic. (b) Mild-warm moxibustion group: the structure of seminiferous tubules is relatively clear, but the shredding of the spermatogenic epithelium remained. (c) Youth control group: the structure of the seminiferous tubules was normal, and the spermatogenic cells at all levels were distributed closely and orderly.

In the youth control group, the structure of the seminiferous tubules was normal. The spermatogenic cells and Sertoli cells at all levels were abundant and distributed tightly and orderly. The different phases of spermatogenic cells could be clearly identified, and the small and round spermatogonia were close to the basal lamina of the seminiferous tubules. Primary spermatocytes, sperm cells, and sperm were orderly distributed in the luminal direction (Figure 1(c)). In the aging group, the spermatogenic cells and Sertoli cells of different phases in the testis were much less abundant, and even mature sperm could hardly be found. The cells were disordered and loose, accompanied by varying degrees of cell deficiency and rupture. The edges of seminiferous tubules were incomplete, and the shapes were irregular. The spermatogenic epithelium was thinned, the number of layers was reduced, and shedding was observed (Figure 1(a)). In the MWMG, the spermatogenic cells and Sertoli cells were distinctly more abundant than in the aging group, and the seminiferous tubule structure was more intact. The spermatogenic cells were neatly distributed throughout the different growth phases. Although there were still cases of spermatogenic epithelial shedding, the structure was close to that of the youth control group (Figure 1(b)).

### 3.2. Moxibustion Enhanced Telomerase Activity of Testis

**Figure 2:**
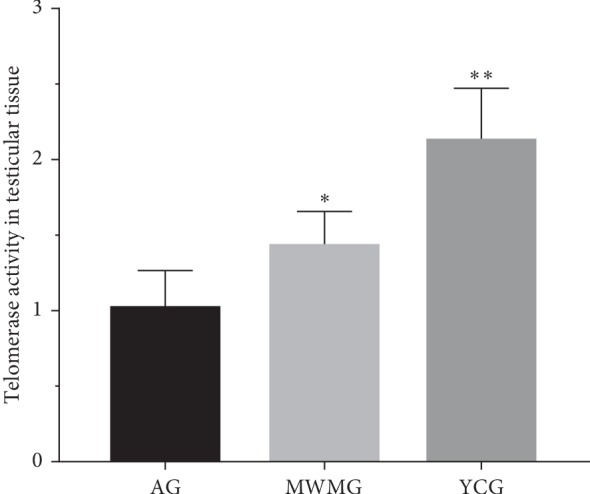
The telomerase activity of the testis is indirectly reflected by the mRNA expression level of TERT detected. AG: aging group, MWMG: mild-warm moxibustion group, YCG: youth control group; each histogram represents mean ± SD (*N* = 8). Compared with the aging group, ^*∗*^*P* < 0.05, ^*∗∗*^*P* < 0.01.

To verify the effectiveness of moxibustion in delaying reproductive senescence, the telomerase activity in the testis was indirectly measured via the mRNA expression of TERT. This study found that telomerase activity in the aging group was very low, while the expression level in the youth control group was significantly higher than that of the aging group (*P* < 0.01). In the MWMG, the telomerase activity of the testis in rats was evidently higher than that of the aging group (*P* < 0.05), however still significantly lower than the level of the youth control group (*P* < 0.01) (Figure 2).

### 3.3. Moxibustion Raised Testosterone Levels

**Figure 3:**
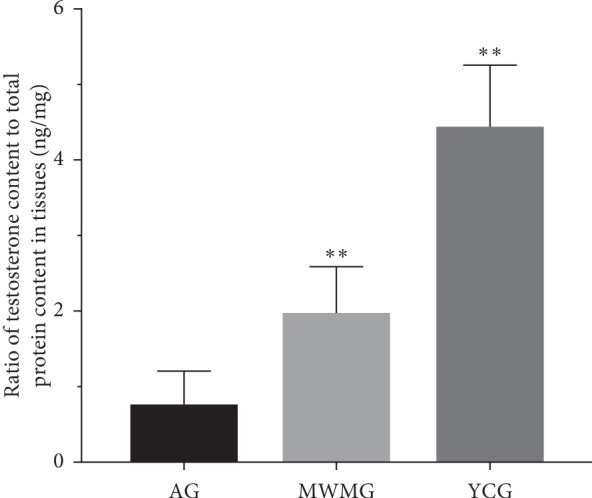
The amount of testosterone contained in the testis (per mg). AG: aging group, MWMG: mild-warm moxibustion group, YCG: youth control group; each histogram represents mean ± SD (*N* = 8). Compared with the aging group, ^*∗∗*^*P* < 0.01.

To explore the impact of moxibustion on gonadal function in rats, we measured testosterone levels in rat testis. Compared with 4.43 ± 0.83 ng of testosterone per mg of testis observed in the youth control group, the testosterone level of the aging group was 0.75 ± 0.47 ng/mg, significantly lower (*P* < 0.01). The MWMG saw testosterone levels significantly raised (*P* < 0.01) to 1.96 ± 0.64 ng/mg (Figure 3).

### 3.4. Moxibustion Improved AR Expression in the Testis

**Figure 4:**
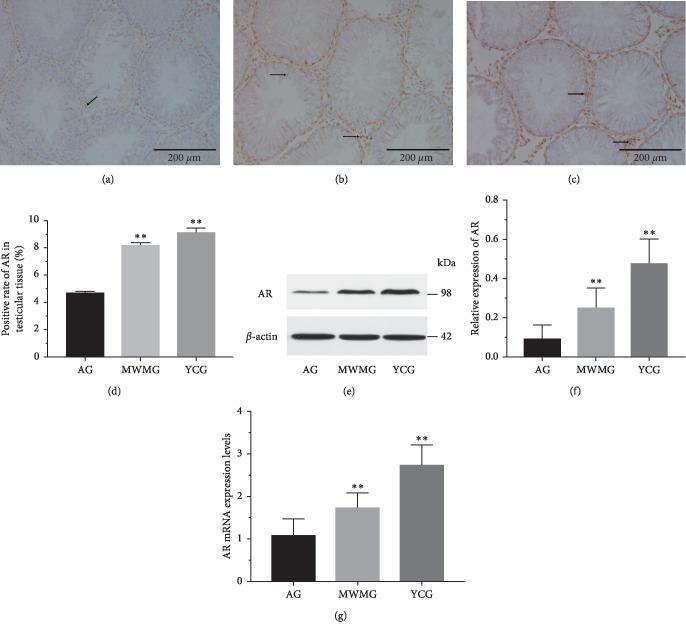
AR expression in the testis of the rat (a–d). Immunohistochemistry was applied to observe and measure the positive expression of AR (bar = 200 *μ*m), and brown particles represented the expression of AR protein. (a) Aging group: less AR-positive expression; almost no expression was in seminiferous tubules, and bits of expression were in the Leydig. (b) Mild-warm moxibustion group: AR-positive expression augmented significantly compared with the aging group, ^*∗∗*^*P* < 0.01; the distribution was similar to the youth control group, though level of expression was less than that of the latter. (c) Youth control group: abundant AR-positive expression primarily concentrated in Sertoli cells, interstitial cells, as well as peritubular myoid cells. In addition, AR expression was observed in VSMC and endothelial cells in the testis. (d) AR positive expression observed by immunohistochemistry. Each histogram represents mean ± SD (*N* = 8). Compared with the aging group, ^*∗∗*^*P* < 0.01. (e–f) Western blot method was applied to measure protein expression of AR. Each histogram represents mean ± SD (*N* = 8). Compared with the aging group, ^*∗∗*^*P* < 0.01. (g) PCR method was applied to measure the mRNA expression of AR. Each histogram represents mean ± SD (*N* = 8). Compared with the aging group, ^*∗∗*^*P* < 0.01. AG: aging group, MWMG: mild-warm moxibustion group, YCG: youth control group.

To explore the impact of moxibustion on the expression of AR in the testis, we observed and measured the protein and mRNA expression level by immunohistochemistry (IHC), western blot assay, and PCR. The experimental results of immunohistochemical analysis (Figure 4) revealed numerous immune-reactive (brown) particles in the youth control group (Figure 4(c)), with the AR-positive expression primarily concentrated in Sertoli cells, interstitial cells, and peritubular myoid cells. AR expression was also observed in vascular smooth muscle cell (VSMC) and endothelial cells of the testis. In contrast, AR expression in the aging group (Figure 4(a)) was significantly lower (*P* < 0.01). In fact, almost no positive AR expression could be found in the seminiferous tubules, though some were found in the Leydig. After moxibustion treatment, the expression of immunopositive cells in the MWMG (Figure 4(b)) multiplied distinctively (*P* < 0.01) compared to the aging group; however, this remained less than that of the youth control group (Figure 4(c)). Interestingly, the distribution of AR-positive cells in the MWMG was similar to that in the youth control group.

To verify the aforementioned experimental results, western blot (Figure 4(e)) assays were carried out. Those results showed that (Figure 4(f)) AR protein expression in the aging group was much lower than that in the youth control group (*P* < 0.01), with AR expression in the aging group totaling about 1/5th of the latter group. Meanwhile, the expression of AR protein increased significantly in the MWMG (*P* < 0.01), corroborating the earlier experiments. Finally, by quantitative PCR method we verified that (Figure 4(g)) mRNA expression of AR in the youth control group was significantly higher than that in the aging group (*P* < 0.01), while mild-warm moxibustion increased aging-related mRNA expression of AR remarkably (*P* < 0.01).

### 3.5. Moxibustion Inhibited Spermatogenic Cell Apoptosis

**Figure 5:**
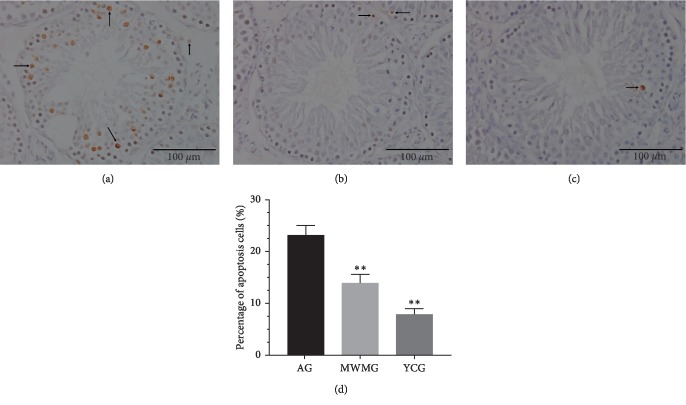
Spermatogenic cell apoptosis (bar = 100 μm). Brown particles represented apoptotic spermatogenic cells. (a) Aging group: the seminiferous tubules observed a large positive reaction, and apoptosis was observed in different growth phases of spermatogenic cells though concentrated in primary spermatocytes and secondary spermatocytes. In comparison, the particles observed in spermatogonia and sperm cells were weakly positive. (b) Mild-warm moxibustion group: the apoptosis rate of spermatogenic cells was low, typical apoptotic cells were few, and some of the particles were weakly positive. (c) Youth control group: almost no apoptotic cells can be observed, and the few positive particles in positive reactions were also considered to be normal physiological apoptosis. (d) The statistics of apoptotic percentage of spermatogenic cells is shown AG: aging group, MWMG: mild-warm moxibustion group, YCG: youth control group; each histogram represented mean ± SD (*N* = 8). Compared with the aging group, ^*∗∗*^*P* < 0.01.

In order to explore the impact of moxibustion on the rate of apoptosis of spermatogenic cells, spermatogenic cell apoptosis was evaluated in three groups by the TUNEL assay. The seminiferous tubules of the aging group were filled with large quantities of immunopositive densities and apoptosis was observed in different growth phases of spermatogenic cells, primarily in spermatocytes and spermatocytes with strong immunopositive signals. By comparison, the immunoreactivity observed in spermatogonia and sperm cells was much weaker, and this level of immunoreactivity was also observed in the Leydig (Figure 5(a)). Almost no apoptotic cells were observed in the youth control group, and the few positive particles were considered normal physiological apoptosis (Figure 5(b)), in contrast to the significantly higher apoptotic signal observed in the aging group (*P* < 0.01). The apoptosis rate of spermatogenic cells in the mild-warm moxibustion group was significantly lower than that in the aging group (*P* < 0.01). Typical apoptotic cells were few in this group, and a small number of weakly positive particles was observed (Figure 5(c)).

### 3.6. Moxibustion Can Regulate the Apoptosis-Related Factors Bax and Bcl-2

**Figure 6:**
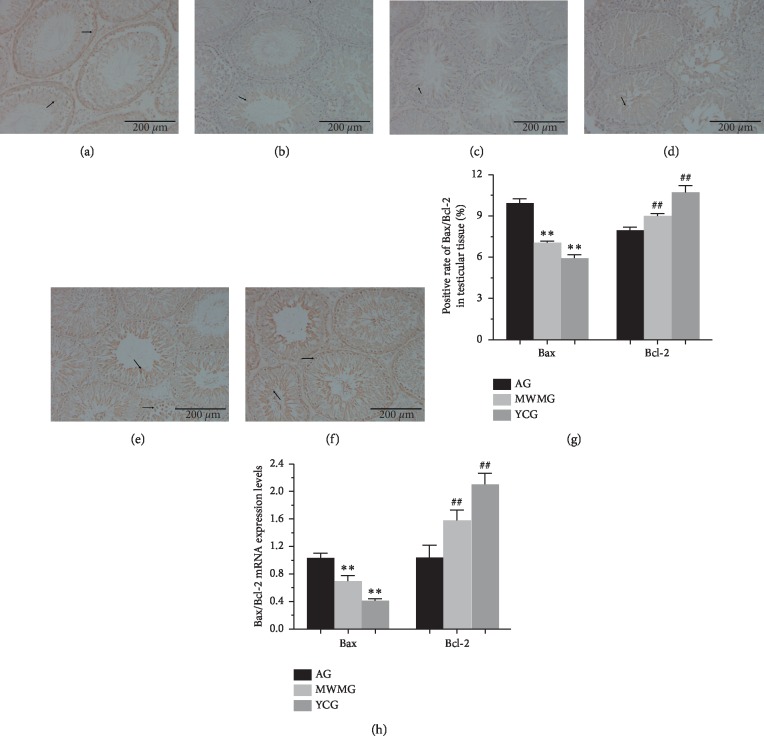
Expression of Bax/Bcl-2 in rat testis (a–c). Immunohistochemistry (IHC) was used to observe and measure the positive expression of Bax (bar = 200 *μ*m), where brown particles represented the expression of Bax protein. (a) Aging group: large quantities of Bax were observed in spermatogenic cells and Leydig at all levels, mainly in spermatogonia and primary spermatocytes. (b) Mild-warm moxibustion group: Bax positive expression was mainly concentrated in sperm cells, but partial positive expression was also observed in spermatogonia, primary spermatocytes, and secondary spermatocytes. (c) Youth control group: only a small amount of positive expression was observed in sperm cells. (d–f) Positive expression of Bcl-2 was observed by immunohistochemistry (bar = 200 *μ*m), and the brown particles represented the expression of Bcl-2 protein. (d) Aging group: Bcl-2 was almost invisible in spermatogonia, and some weakly positive reactions were concentrated in sperm cells. (e) Mild-warm moxibustion group: the seminiferous tubules were filled with large quantities of spermatogenic cells expressing Bcl-2, and only bits of the cells in the Leydig were not positively expressed. (f) Youth control group: almost all cells were in positive reaction. (g) Statistics of immunohistochemical positive expression rate in terms of Bax and Bcl-2. Each histogram represents mean ± SD (*N* = 8). Compared with the aging group, ^*∗∗*^*P* < 0.01, ^##^*P* < 0.01 (h) mRNA expression of Bax/Bcl-2 was measured by the PCR assay. AG: aging group, MWMG: mild-warm moxibustion group, YCG: youth control group; each histogram represents mean ± SD (*N* = 8). Compared with the aging group, ^*∗∗*^*P* < 0.01, ^##^*P* < 0.01.

In order to explore the impact of moxibustion on the expression of apoptosis-related factors Bax and Bcl-2, we observed and measured the protein expression of these factors by immunohistochemistry and measured mRNA expression by PCR. From the results of immunohistochemical assessments, large quantities of Bax were found in spermatogenic cells and Leydig. While this was apparent during all growth phases, Bax was mainly concentrated in spermatogenic cells and primary spermatocytes (Figures 6(a), 6(b) and 6(g)). In contrast, the positive expression of Bax in the youth control group was significantly lower (*P* < 0.01) (Figures 6(c) and 6(g)), with only a small amount of Bax expression detected in the sperm cells, with weakly positive expression also observed in spermatogonia, primary spermatocytes, and secondary spermatocytes. In contrast, almost no Bcl-2 immunoreactivity was detected in the spermatogonia of the aging group (Figures 6(d), 6(e), and 6(g)), though some weakly positive reactions were observed in sperm cells. Bcl-2 expression in the youth control group was, by comparison, noticeably higher (*P* < 0.01) (Figures 6(f) and 6(g)), with positive expression found in almost all cell types. The expression of Bcl-2 in the MWMG was significantly higher than in the aging group (*P* < 0.01). In the MWMG the seminiferous tubules were filled with large quantities of detectable Bcl-2 in spermatogenic cells, while only portions of cells in the Leydig were devoid of Bcl-2. Subsequently, PCR verification demonstrated that Bax mRNA expression in the youth control group was significantly lower than that in the aging group (*P* < 0.01), while the mRNA expression of Bcl-2 was significantly higher (*P* < 0.01). The moxibustion experimental group displayed reduced mRNA expression of Bax (*P* < 0.01) and augmented expression of Bcl-2 mRNA compared to the aging group (Figure 6(h)).

## 4. Discussion

Aging is an irreversible trend normally occurring in biological organisms after growth to maturity. The overall systematic capacity of the body gradually decreases as males age, including the *in vivo* environmental stability and stress response capacity of the human body and the integrity and function of tissue architecture, which degrades gradually and eventually leads to death [11]. The theory in the traditional Chinese medical classic “The Yellow Emperor's Classic of Medicine” has been used for thousands of years as a guide to prevent disease, nurture life, and delay senescence. The text creatively proposed the concept of inconsistencies in the aging process between men and women, largely caused by gonadal differences. In a similar vein, modern medical research has highlighted that with age, and gene expression across different tissues is gradually altered from spatial synchronization to spatial asynchrony, leading to the eventual discoordination of tissues. For example, compared to the female rat, the gene expression of the male rat demonstrates symptoms of spatial asynchrony much faster, indicating that male animals are prone to biological aging more rapidly [12]. Therefore, understanding the mechanisms underlying the male aging process is of great significance.

A prominent feature of aging is the deterioration of tissue morphology and structure, which affects both the spermatogenic function and androgen producing function of the testis [13]. Through stereological analysis, it was found by Miranda et al. [14] that the typical manifestation of rat aging includes a significant reduction in the number of sperm cells and the area of seminiferous tubules. Similar results have been confirmed in human experiments [15]. A well-established conclusion is that all testicular functions are interrelated and that testosterone levels play an important role in spermatogenesis and maturation [16]. Therefore, the decrease in testosterone level caused by aging may be accountable for the decline of spermatogenic function, though it is by no means the only cause. Interestingly, it has been reported that the symptoms caused by this decrease in testosterone are not entirely related to testosterone levels as experimentally manipulated [17]. In view of the aforementioned scenario, we proposed that simple T supplementation therapy may have limited healing efficacy on the overall recession of male gonadal function caused by aging, wherein other risk factors may arise [18].

Moxibustion is a technique derived from traditional medical practice of the ancient Chinese. It has been widely applied for the treatment of various diseases and is believed to function through the effects of meridian warming and cold dispelling, reinforcement and relieving depletion of Yang Qi, blood stasis, and elimination of hard lumps, culminating in a wealth of disease prevention. Modern research has confirmed that moxibustion plays an important role in delaying aging and regulating bodily function [19–21]. Researchers have also confirmed that mild-warm moxibustion can raise testosterone levels in aging rats, regulate their immune function, and improve the health status of older rats [22]. On the other hand, the research evidence remains insufficient, and the proposed mechanisms are superficial, warranting deeper empirical studies.

In this experiment, moxibustion improved the testosterone level in the testis of aging rats, which was rarely studied in the past. It supports that moxibustion has a therapeutic efficacy on low level testosterone caused by aging. This efficacy is not only manifested in the functional aspect but also in the improvement of the testicular tissue architecture. The structure of the seminiferous tubules after moxibustion appears more distinctive, and the distribution of spermatogenic cells is generally more organized.

It is widely acknowledged that the degree of senescence can be evaluated by telomere length, as telomere length decreases with aging, acting as one of the main sources for cellular senescence as verified by the inversely proportional relationship between telomere length and age [23, 24]. Although telomere length and its depletion rate vary greatly from one individual to another, it is still widely believed that before adulthood, telomere length is stable, decreasing with age after that [25]. In addition, Anchelin et al. [26] confirmed that a direct correlation exists among telomere length and testicular function and age. Telomere length and longevity can therefore be retained, and testicular function can be improved by the restoration of telomerase activity. Similar results were verified in our experiments. The telomerase activity in the testis of the aging group was significantly lower than that of the youth control group, and mild-warm moxibustion enhanced telomerase activity and delayed the reproductive senescence.

Apoptosis is a normal biological process that regulates the growth and development of living organisms to maintain homeostasis [27]. Similarly, apoptosis plays an important role in cellular differentiation, sperm maturation, and survival of the testis. On the one hand, germ cells with genetic defects need to be removed effectively to ensure that defects will not be transmitted to the offspring [28]. On the other hand, the balance between different cells can be maintained by this process, which is essential to spermatogenesis and male reproduction [29]. Aging ultimately leads to a remarkable augmentation of testicular germ cell apoptosis, which is usually observed through primary spermatocytes and the prominently declining number of sperm cells [30]. TUNEL assay results in this study showed that the seminiferous tubules in the aging group were filled with large quantities of apoptotic cells, concentrated in the primary spermatocytes and secondary spermatocytes. This is consistent with the previous literature [30, 31]. In addition, a positive reaction signaling apoptosis was observed in the Leydig. As demonstrated here, mild-warm moxibustion can significantly reduce the apoptosis rate of spermatogenic cells, mainly inhibiting apoptosis at the phase of the primary spermatocytes.

The mitochondrial channel is an important endogenous channel in the process of spermatogenic cells apoptosis. When cells are exposed to an apoptotic stimulator, they activate intracellular signals to induce apoptosis [32–34]. In the testis, mitochondrial dysfunction can be exacerbated, and mitochondrial impairment can be aggravated by T decrease [35]. The regulation of mitochondrial permeability transition pores (MPTP) by Bcl-2 family of proteins holds the key to this process [36]. Only when MPTP is open can a series of proapoptotic substances including cytochrome C (CytC) be released from the mitochondria into the cytoplasm [37], triggering apoptosis after substrate cleavage by proapoptotic substances induced by the cascade signaling of activated caspases [38]. The opening of the MPTP is induced by oligomerization permitted by the proapoptotic protein Bax (a member of the Bcl-2 family), an irreversible process of apoptosis. Alternatively, the antiapoptotic protein Bcl-2 can inhibit the opening of MPTP by binding to Bax directly [39]. Therefore, the Bcl-2 family proteins can maintain an equilibrium state through the interaction of antiapoptotic proteins with proapoptotic proteins, thereby regulating the permeability of mitochondrial outer membrane. Therefore, Bcl-2/Bax ratios are key determinants of the opening of the MPTP. When cells are stimulated by various factors, apoptosis can be directly determined by the ratio of antiapoptotic protein and proapoptotic protein [40]. In this study, immunohistochemistry and PCR were applied to detect the expression levels of these factors. It was indicated that the expression of Bax protein in aging rats was significantly higher than that in the youth control group, while mild-warm moxibustion could significantly reduce this expression. In contrast, Bcl-2 expression of aging rats was significantly lower than that in the youth control group, and mild-warm moxibustion could increase this expression remarkably. These results indicate that mild-warm moxibustion can inhibit the apoptosis of germ cells in the testis by normalizing the levels of Bcl-2/Bax.

Although it is now widely recognized that AR expression is critical for maintaining normal function of male germ cells [41], its normal expression level remains a subject of controversy. On the one hand, the report of an absence of AR in testicular germ cells has been inconsistent among experts [42–45]. In general, it is believed that T cannot affect germ cells directly, rather it creates a microenvironment suitable for spermatogenesis by acting on Sertoli cells and peritubular myoid cells, a notion supported by this study. On the other hand, whether or not the expression level of AR is altered with age remains a topic for future discussion. At present, many researchers still believe that the expression of AR has nothing to do with age [46, 47]. But we think they are relevant. Since the reproductive regulation of testosterone needs to combine with AR-expressing cells in order to complete transcription, translation, and other processes to ensure spermatocyte survival and meiosis, reproductive senescence cannot be independent of AR. Moreover, in consideration of the profound effect of AR on nervous, immune, cardiovascular, and metabolic systems closely related to aging [48], we assume that the expression of AR in the testis would decline with age, which is consistent with the views illustrated in some scholarly articles [49–51]. The quantitative and semiquantitative studies of AR expression in this study were conducted via immunohistochemistry, PCR, and western blot assay, the results of which indicated that the AR expression of rats in the aging group was significantly lower than that in the youth control group, which confirmed our hypothesis and that mild-warm moxibustion could improve its expression perceivably.

## 5. Conclusion

In summary, mild-warm moxibustion can raise the T level in the testis of aging rats, repair the impaired testicular tissue architecture caused by aging, protect the seminiferous tubule tissue structure, delay general tissue aging, improve the expression of AR in the testis by enhancing telomerase activity, and inhibit the apoptosis of spermatogenic cells, the latter of which was found to be regulated at least partly by raising Bcl-2/Bax via mitochondrial channels.

## Figures and Tables

**Table 1:** The primers of Bax, Bcl-2, and *β*-actin.

Gene	Primer sequences	Annealing Tm (°C)	Product size (bp)
*Bax*	F : AGACAGGGGCCTTTTTGCTAC	60	147
	R : AGACAGGGGCCTTTTTGCTAC		

*Bcl-2*	F : TGGAGGAACTCTTCAGGGATGG	60	183
	R : CATCCCAGCCTCCGTTATCC		

*AR*	F : AGACAGGGGCCTTTTTGCTAC	60	278
	R : AGACAGGGGCCTTTTTGCTAC		

*β-actin*	F : GACGTTGACATCCGTAAAGACC	60	125
	R : TGCTAGGAGCAGGGGTA		

## Data Availability

The data used to support the findings of this study are available from the corresponding author upon request.

## Abbreviations

AR: Androgen receptor
BL23: Shenshu acupuncture points
MPTP: Mitochondrial permeability transition pores
MWMG: Mild-warm moxibustion group
ST36: Zusanli acupuncture points
TERT: Termed telomerase reverse transcriptase
DAB: Diaminobenzaidine
VSMC: Vascular smooth muscle cell
MPTP: Mitochondrial permeability transition pores
CytC: Cytochrome C
T: Testosterone.

## Additional Points

Age-related decline in telomerase and testosterone was improved by mild-warm moxibustion. Mild-warm moxibustion inhibited spermatogenic apoptosis and improved testicular structure. Mild-warm moxibustion alleviated reproductive senescence in part by regulation of Bax/Bcl-2.
